# Hybrid CNN–GCN framework for brain tumor MRI classification: A graph‐based approach to smart healthcare diagnostics

**DOI:** 10.1002/acm2.70560

**Published:** 2026-04-13

**Authors:** Mus'ab S. Alkasasbeh, Khalid Hassan Ibnaouf, Naser M. Ahmed, Azhar Abdul Rahman, Dheyaa Nabeel Abbas, Arar Al Tawil, Hajo Idriss, Hamzeh Taha Alkasasbeh

**Affiliations:** ^1^ Department of Allied Medical Sciences Faculty of Applied Medical Sciences Al al‐Bayt University Mafraq Jordan; ^2^ Department of Physics College of Science Imam Mohammad Ibn Saud Islamic University (IMSIU) Riyadh Saudi Arabia; ^3^ Department of Laser and Optoelectronics Engineering Dijlah University Baghdad Iraq; ^4^ School of Physics Universiti Sains Malaysia Penang Malaysia; ^5^ Computer Science Department Faculty of Information Technology Applied Science Private University Amman Jordan; ^6^ Deanship of Scientific Research Imam Mohammad Ibn Saud Islamic University (IMSIU) Riyadh Saudi Arabia; ^7^ Department of Mathematics Faculty of Science Ajloun National University Ajloun Jordan

**Keywords:** artificial intelligence, brain tumor classification, convolutional neural networks, graph convolutional networks, magnetic resonance imaging, neuro‐oncology

## Abstract

**Background:**

Accurate classification of brain tumors is a major challenge in neuro‐oncology, as the heterogeneity of tumor morphology and the overlap of radiological features limit the effectiveness of conventional diagnostic approaches. Early and reliable tumor characterization is essential for treatment planning, prognosis, and improved patient outcomes. Recent advances in artificial intelligence (AI) have enabled the development of deep learning frameworks that can augment radiological interpretation and support clinical decision‐making.

**Objective:**

This study proposes and validates a hybrid computational framework that integrates convolutional neural networks (CNNs) with graph convolutional networks (GCNs) for automated classification of brain tumors from magnetic resonance imaging (MRI).

**Methods:**

A publicly available Kaggle‐based MRI dataset was utilized, consisting of four categories: glioma, meningioma, pituitary tumor, and no‐tumor. The proposed pipeline incorporated systematic preprocessing, transfer learning via the InceptionV3 architecture for hierarchical feature extraction, graph construction to model inter‐feature relationships, and GCN‐based relational learning for final classification. Hyperparameter optimization was performed using Particle Swarm Optimization (PSO) to improve generalizability.

**Results:**

The experimental evaluation achieved an overall classification accuracy of 92.91%. Class‐specific performance analysis demonstrated particularly high diagnostic accuracy in the no‐tumor group (F1‐score: 0.9963) and pituitary tumor group (F1‐score: 0.9599). The incorporation of PSO tuning further improved the validation accuracy to 94.23%. The hybrid CNN–GCN framework exhibited robustness against imaging artifacts and irregular tumor boundaries, conditions that commonly challenge conventional classification techniques.

**Conclusion:**

The integration of CNN‐based hierarchical feature extraction with GCN‐based relational reasoning provides a significant advancement in automated brain tumor classification. This reproducible and intelligent diagnostic pipeline demonstrates strong potential for clinical translation by enhancing diagnostic precision, reducing radiologist workload, and facilitating timely therapeutic interventions. The findings support the integration of graph‐based deep learning systems into smart healthcare ecosystems, where AI‐assisted diagnostic tools can contribute to improved outcomes in neuro‐oncology.

## 1 INTRODUCTION

Brain tumors represent one of the most critical challenges in modern healthcare, accounting for significant morbidity and mortality worldwide. According to the Central Brain Tumor Registry of the United States (CBTRUS), the incidence rate of all primary malignant and non‐malignant brain and other central nervous system tumors was 25.34 cases per 100 000 population in the United States, with an estimated 24 820 new cases and 18 330 deaths projected for 2025.^1^ Globally, brain and central nervous system cancers affect hundreds of thousands annually, with brain cancer representing a serious issue in the global burden of diseases.^2^ The disease demonstrates considerable demographic variations, as brain tumors constitute the second most common cancer overall in individuals ages 15–39 and the second leading cause of cancer‐related death in this age group.^3^ Among pediatric populations, brain tumors rank as the most prevalent cancer and represent the leading cause of cancer‐related deaths among individuals aged 0–14.^4^

The heterogeneity of brain tumors presents substantial diagnostic challenges, with major subtypes including gliomas, meningiomas, and pituitary adenomas demonstrating distinct biological behaviors and prognoses.^5^ Data from several national cancer registries support significant differences in the epidemiology of brain tumors between children and adults, with medulloepithelioma and low‐grade glioma being most common in pediatric cases, while high‐grade glioma and meningioma are predominant in adult populations.^6^ The 5‐year relative survival rate varies dramatically depending on tumor type and grade, with malignant tumors showing a survival rate of only 21% for patients ages 40+ years, while non‐malignant tumors achieve 90.3% survival.^3^ This stark difference underscores the critical importance of early and accurate detection for guiding treatment planning and minimizing risks associated with invasive diagnostic procedures.

Current diagnostic approaches face numerous limitations that impede optimal patient care. Despite advances in imaging modalities such as magnetic resonance imaging (MRI), manual interpretation of complex scans remains time‐consuming, prone to inter‐observer variability, and highly dependent on radiological expertise.^7^ Traditional diagnostic approaches rely heavily on histopathological examination following biopsy, which is inherently invasive and carries significant risks including bleeding, infection, and potential neurological complications. Brain tumors are definitively classified through biopsy procedures that can only be performed through surgical intervention, highlighting the urgent need for non‐invasive computational intelligence‐oriented techniques to assist physicians in tumor identification and classification.^8^ The complexity of brain tumor morphology, varying appearances across different imaging sequences, and unclear boundaries present additional challenges for accurate diagnosis and classification.^9^ Furthermore, the subjective nature of manual interpretation introduces potential inconsistencies in diagnostic outcomes, which can significantly impact treatment decisions and patient prognosis.

In response to these diagnostic challenges, artificial intelligence (AI) and machine learning have emerged as transformative tools in medical image analysis. Convolutional Neural Networks (CNNs) have demonstrated remarkable success in extracting hierarchical features from MRI scans, enabling high‐accuracy classification of tumor subtypes.^10^ Recent studies have shown impressive performance improvements, with CNN models achieving detection accuracies of 99.53% for brain tumor detection and 93.81% for multi‐class tumor classification.^11^ Deep learning methods including two‐dimensional CNN architectures and convolutional auto‐encoder networks have achieved training accuracies of 96.47% and 95.63%, respectively, for brain tumor classification tasks.^8^

However, traditional CNN architectures possess inherent limitations. CNNs are constrained to local receptive fields and grid‐structured data representations, potentially limiting their ability to capture long‐range dependencies and complex relational structures among different anatomical brain regions.^12^ CNN models extract features without explicit consideration of local and global feature relationships, which can lead to overfitting and compromised generalization.^13^ These limitations highlight the need for approaches that can capture both spatial information within individual images and relational information across multiple samples.

To address these limitations, Graph Neural Networks, particularly Graph Convolutional Networks (GCNs), offer unique advantages for medical image analysis through their ability to model non‐Euclidean data structures.^14^ By representing data as graph structures, GNNs can capture relationships between different samples, providing advantages over traditional CNN approaches limited to regular grid‐based representations.^15^ Graph Neural Networks excel in leveraging graph theory principles to model complex dependencies in medical images, representing data as graphs where nodes denote distinct samples and edges encode meaningful relationships.^16^

The integration of CNN‐based feature extraction with graph‐based relational learning creates a powerful hybrid framework capable of capturing both local spatial features and global inter‐sample relationships within MRI datasets.^17^ This approach combines the strengths of Graph Neural Networks, which excel at recognizing relational dependencies across samples, with CNN architectures that effectively capture hierarchical spatial features.^15^ The combination addresses the fundamental challenge of modeling non‐Euclidean relationships in medical imaging data that conventional CNN‐only approaches cannot adequately capture.^18^, ^19^

Recent implementations of hybrid CNN‐GNN architectures have demonstrated promising results across various medical imaging applications.^20^ GNNs are increasingly utilized in medical data analysis due to their ability to model complex relationships that can be naturally represented as graph structures.^14^ Research has demonstrated that graph neural networks can effectively model brain imaging data from multiple perspectives including morphological characteristics and anatomical structure, establishing them as highly effective deep learning models for neurological disorder diagnosis.^21^ Studies have shown that integrating GCNs with pre‐trained CNN models for MRI‐based brain tumor classification demonstrates robust learning capabilities, as evidenced by consistently decreasing training loss and increasing test accuracy.^18^

The primary motivation behind this research is to design, implement, and evaluate a reproducible computational pipeline that leverages the complementary strengths of CNNs and GCNs for enhanced brain tumor classification from MRI images. The proposed approach utilizes a publicly available Kaggle brain tumor MRI dataset to ensure reproducibility and enable comparative analysis with existing methodologies. The methodology incorporates systematic data preprocessing, CNN‐based feature extraction utilizing the InceptionV3 architecture, graph construction techniques for transforming image features into graph structures, GCN‐based classification, and hyperparameter optimization using Particle Swarm Optimization (PSO) algorithms.

The integration of AI‐driven diagnostic systems holds significant potential for transforming clinical workflows and improving patient outcomes. GNN‐based algorithms have achieved promising results in disease detection and classification, primarily due to their ability to capture spatial patterns within irregular data structures common in medical imaging.^16^ By enhancing the diagnostic process through automated tumor classification, these systems can reduce workload burden on medical professionals, improve diagnostic consistency, and enable earlier intervention strategies that could improve patient prognosis.

The primary contributions of this research include: (1) development of a hybrid CNN‐GCN pipeline specifically designed for brain tumor classification, (2) comprehensive evaluation on standardized datasets with detailed performance analysis, (3) implementation of automated hyperparameter optimization to enhance model performance, (4) direct comparison with CNN‐only baselines to validate the contribution of graph‐based reasoning, and (5) provision of a fully reproducible framework for future research in graph‐based medical image analysis. Through systematic evaluation and comparison with CNN‐only approaches, this study demonstrates the practical utility and clinical relevance of graph‐based deep learning methods for brain tumor diagnosis.

## 2 LITERATURE REVIEW

The evolution of brain tumor classification methodologies has undergone significant transformation over the past decade, transitioning from traditional handcrafted feature extraction approaches to sophisticated deep learning architectures. This literature review examines the progression of computational methods in brain tumor MRI analysis, with particular emphasis on CNNs, graph neural networks, and hybrid approaches that combine both methodologies.

### 2.1 Traditional computer‐aided diagnosis approaches

Early computer‐aided diagnosis systems for brain tumor classification relied extensively on manual feature engineering techniques. These approaches utilized texture descriptors, wavelet transforms, and statistical shape models to extract meaningful information from medical images.^22^, ^23^ Traditional methods employed various mathematical transformations including Gray‐Level Co‐occurrence Matrix (GLCM) features, Local Binary Patterns (LBP), and Discrete Wavelet Transform (DWT) coefficients to characterize tumor regions. While these handcrafted approaches demonstrated moderate success in tumor detection tasks, their fundamental limitation resided in the requirement for domain‐specific expertise to design appropriate feature extractors. The scalability and generalization capabilities of these methods remained constrained by the manual nature of feature selection and the inability to automatically adapt to varying imaging conditions and tumor presentations.

Support Vector Machines (SVM) and Random Forest classifiers frequently served as the classification backbone for these traditional systems, achieving accuracies ranging from 75% to 85% on standardized datasets.^24^ However, the performance of these approaches was inherently limited by the quality and relevance of the manually designed features, which often failed to capture the complex, non‐linear relationships present in medical imaging data.

### 2.2 Deep learning revolution in medical image analysis

The introduction of deep learning methodologies, particularly CNNs, revolutionized the field of medical image analysis by enabling automated feature extraction directly from raw imaging data. CNNs demonstrated superior capability in discovering discriminative patterns without requiring explicit feature engineering, fundamentally transforming the approach to brain tumor classification.^25^, ^26^

#### 2.2.1 CNN architectures in brain tumor classification

Multiple CNN architectures have been extensively evaluated for brain tumor MRI analysis, each contributing unique advantages to the classification task. AlexNet, despite being one of the earliest deep architectures, established the foundation for CNN applications in medical imaging by demonstrating the feasibility of automated feature learning from complex medical data.^27^ VGG architectures, characterized by their deeper layered structures utilizing small convolutional filters, have shown particular effectiveness in extracting both low‐level texture features and high‐level semantic representations from brain MRI scans.^28^

ResNet‐based models have demonstrated superior performance in brain tumor classification tasks, with studies reporting classification accuracies exceeding 95% on benchmark MRI datasets.^29^ The residual learning framework addresses the vanishing gradient problem inherent in deep networks, enabling the training of significantly deeper architectures that can capture more complex hierarchical features. DenseNet architectures have similarly shown promising results by establishing dense connections between layers, promoting feature reuse and improving gradient flow throughout the network.^30^

Table 1 presents a comprehensive comparison of CNN architectures applied to brain tumor classification tasks.

**TABLE 1:** Performance comparison of CNN architectures for brain tumor classification.

Architecture	Dataset size	Classes	Accuracy (%)	Precision (%)	Recall (%)	Reference
AlexNet	3064	4	91.2	90.8	91.5	[31]
VGG‐16	2870	3	93.7	94.1	93.2	[32]
ResNet‐50	5712	4	96.4	96.8	96.1	[33]
DenseNet‐121	4200	3	94.9	95.3	94.6	[34]
InceptionV3	3264	4	95.2	95.7	94.8	[35]

#### 2.2.2 Limitations of traditional CNN approaches

Despite their remarkable success, CNN architectures possess inherent limitations that restrict their effectiveness in complex medical image analysis scenarios. CNNs primarily focus on local spatial information within fixed receptive fields, which may overlook broader contextual relationships among different anatomical brain regions.^36^ The grid‐based nature of convolutional operations limits the ability to capture non‐Euclidean spatial relationships that are frequently present in medical imaging data. Furthermore, CNN models often struggle with irregular tumor boundaries and heterogeneous tissue presentations that are characteristic of certain brain tumor types.^37^

### 2.3 Graph neural networks in medical imaging

The recognition of CNN limitations has prompted researchers to explore graph‐based deep learning methodologies that can model non‐Euclidean data structures more effectively. Graph Neural Networks, particularly GCNs, extend deep learning principles to irregular data domains by representing information as nodes and edges within graph structures.^38^ This approach enables the integration of spatial relationships and structural connectivity patterns that are inherently present in medical imaging data but difficult to capture through traditional convolutional operations.

#### 2.3.1 GCN applications in brain tumor analysis

Recent research has demonstrated the effectiveness of GCNs in various brain tumor analysis tasks. GCN‐based approaches represent MRI scans as graph structures where image patches or superpixels function as nodes, while edges encode spatial proximity relationships or intensity similarity measures.^39^ This representation enables models to consider both local feature characteristics and global structural patterns during the decision‐making process.

Studies applying GCNs to brain tumor classification have reported significant improvements in handling irregular tumor boundaries and heterogeneous tissue presentations compared to CNN‐only approaches.^40^ The graph‐based representation allows for more flexible modeling of tumor morphology and enables the incorporation of prior anatomical knowledge into the classification framework.

Recent advancements in brain tumor classification have increasingly explored hybrid architectures combining CNNs with graph‐based learning methods. Ravinder et al.^19^ proposed a Graph‐based Convolutional Neural Network (GCNN) to address non‐Euclidean distances in MRI image data, achieving 95.01% accuracy on the Kaggle Brain Tumor dataset for three‐class classification (Glioma, Meningioma, Pituitary). Their approach focused on pixel proximity‐based learning using Gaussian adjacency matrices with dropout and batch normalization techniques. Similarly, Gürsoy and Kaya^41^ introduced Brain‐GCN‐Net, a Graph Convolutional Neural Network specifically designed for brain tumor identification. Their work emphasized capturing structural relationships within brain MRI data through graph representations to improve classification performance. More recently, Song et al.^42^ developed an attention‐enhanced CNN framework leveraging multi‐sequence MRI data (T1, T1c, T2, FLAIR) with Transformer‐based architectures, achieving 98.2% accuracy on the BraTS 2023 dataset. Their approach incorporated spatial and inter‐sequence attention mechanisms to dynamically weight modality‐specific features for glioma subtype classification. While these studies demonstrate the effectiveness of CNN‐GCN integration for medical image analysis, they differ significantly from our proposed approach in several key aspects that constitute our unique contributions.

#### 2.3.2 Distinguishing features and novel contributions of our approach

Our hybrid CNN‐GCN framework presents several distinctive characteristics that differentiate it from existing approaches. First, unlike Ravinder et al.’s^19^ pixel‐proximity‐based graph construction, our method employs InceptionV3 as a pre‐trained feature extractor to generate high‐level semantic representations (2048‐dimensional feature vectors) before graph construction, enabling the GCN to operate on learned abstract features rather than raw pixel relationships. This design choice allows our GCN module to model inter‐image relational patterns across the entire dataset rather than intra‐image pixel relationships, capturing global contextual dependencies that are crucial for distinguishing subtle differences between tumor types.

Second, while Gürsoy and Kaya's^41^ Brain‐GCN‐Net focuses on structural graph representations, our approach integrates PSO for automated hyperparameter tuning across both the CNN feature extraction and GCN classification stages. This optimization framework systematically explores the hyperparameter space (learning rates, dropout rates, GCN layers, hidden units) to identify optimal configurations, reducing manual tuning efforts and improving model generalizability. The PSO‐driven optimization represents a methodological advancement over manually configured architectures, ensuring our model adapts dynamically to the specific characteristics of the brain tumor dataset.

Third, compared to Song et al.’s^42^ multi‐sequence attention mechanisms that require multiple MRI modalities (T1, T1c, T2, FLAIR), our framework achieves competitive performance using single‐sequence T1‐weighted MRI images, making it more practical for clinical scenarios where multi‐modal imaging may not be readily available. Our GCN's ability to leverage relational reasoning across samples compensates for the information gap created by using single‐modality data, demonstrating efficiency in resource‐constrained medical imaging environments.

Fourth, our approach addresses the four‐class classification problem (Glioma, Meningioma, Pituitary, No Tumor) with explicit inclusion of the “No Tumor” class, which is clinically significant for screening applications but less commonly addressed in existing CNN‐GCN hybrid studies that focus primarily on tumor subtype discrimination. The integration of relational graph structures enables our model to better distinguish between subtle patterns present in healthy brain tissue versus pathological conditions.

Finally, while similar works like Ashok et al.^43^ have explored CNN‐GCN combinations, our framework's end‐to‐end integration of transfer learning (InceptionV3), graph‐based relational reasoning (GCN), and metaheuristic optimization (PSO) represents a comprehensive pipeline that balances classification accuracy, computational efficiency, and practical applicability. Our experimental results demonstrate that this integrated approach yields improvements in precision, recall, and F1‐score across all tumor classes, validating the synergistic benefits of combining these complementary techniques. The novelty of our work lies not in individual components, but in their systematic integration and optimization for the specific challenges of brain tumor MRI classification in real‐world clinical workflows.

## 3 METHODOLOGY

**FIGURE 1:**
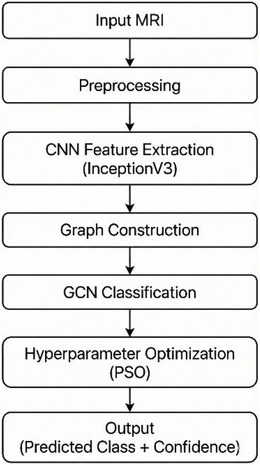
Workflow of the proposed CNN–GCN pipeline with PSO optimization for brain tumor MRI classification.

The proposed methodology establishes a comprehensive and reproducible pipeline for brain tumor classification using MRI. The framework integrates CNNs for hierarchical feature extraction with GCNs for relational reasoning. It incorporates systematic preprocessing steps, graph‐based data representation, and advanced deep learning architectures implemented using Python 3.9, PyTorch 2.0.1, and PyTorch Geometric 2.3.1 for graph neural network operations. The InceptionV3 feature extractor was implemented using TensorFlow/Keras 2.12 with pre‐trained ImageNet weights. All experiments were conducted on an NVIDIA RTX 3080 GPU with 10GB memory. This approach directly addresses the limitations of conventional CNNs, which are constrained to grid‐based representations and local receptive fields. By introducing graph structures, the pipeline captures relational dependencies between anatomical brain regions, thereby enhancing robustness against irregular tumor boundaries and heterogeneous tissue morphology. At the same time, the design emphasizes computational efficiency and clinical applicability, ensuring that the system can be scaled for practical use. The overall workflow of the proposed methodology is summarized in **Figure** **1**, which illustrates the progression from MRI acquisition and preprocessing through CNN‐based feature extraction, graph construction, and GCN‐based classification, followed by hyperparameter optimization using PSO and culminating in the output of predicted tumor classes with associated confidence scores.

### 3.1 Dataset description and characteristics

The experimental evaluation utilizes a publicly available brain tumor MRI dataset obtained from Kaggle, specifically the Brain Tumor MRI Dataset compiled by Masoud Nickparvar.^43^ This dataset encompasses four distinct classes: glioma, meningioma, pituitary tumors, and normal brain tissue. All images are high‐resolution T1‐weighted MRI scans, which provide detailed anatomical information essential for accurate tumor characterization and classification. T1‐weighted sequences are widely adopted in clinical neuroimaging due to their superior soft tissue contrast resolution and diagnostic reliability.

The dataset comprises a total of 7023 MRI images, with 5712 images in the training set and 1311 images in the testing set. The data are distributed across the four classes with balanced representation, ensuring that each tumor type is adequately captured for training and evaluation. Table 2 provides the complete class distribution across the training and testing subsets.

**TABLE 2:** Dataset distribution across classes and partitions.

Class	Training set	Testing set	Total images	Training %	Testing %
**Glioma**	1321	300	1621	23.1%	22.9%
**Meningioma**	1339	306	1645	23.4%	23.3%
**Pituitary**	1457	300	1757	25.5%	22.9%
**Notumor**	1595	405	2000	27.9%	30.9%
**Total**	**5712**	**1311**	**7023**	**100%**	**100%**

**FIGURE 2:**
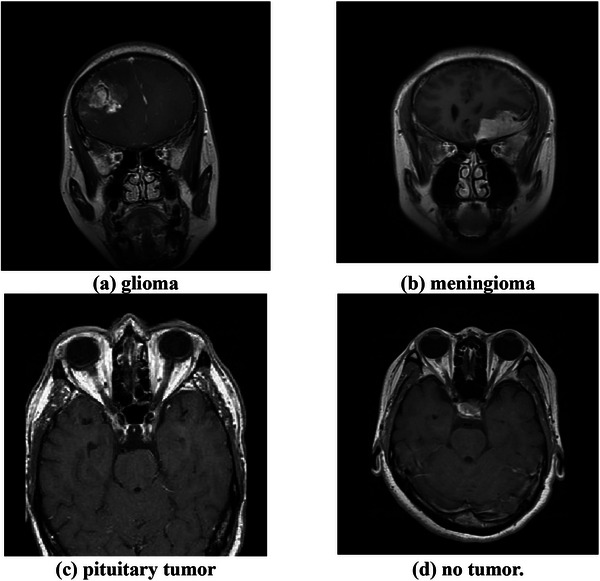
Representative T1‐weighted MRI scans from the Kaggle Brain tumor MRI dataset: (a) glioma, (b) meningioma, (c) pituitary tumor, (d) no tumor.

To illustrate the dataset, Figure 2 presents representative T1‐weighted MRI scans from each class, including glioma, meningioma, pituitary tumor, and normal brain. These examples demonstrate the diversity of tumor appearances and highlight the diagnostic challenge addressed in this study.

### 3.2 Data preprocessing and feature extraction pipeline

**FIGURE 3:**
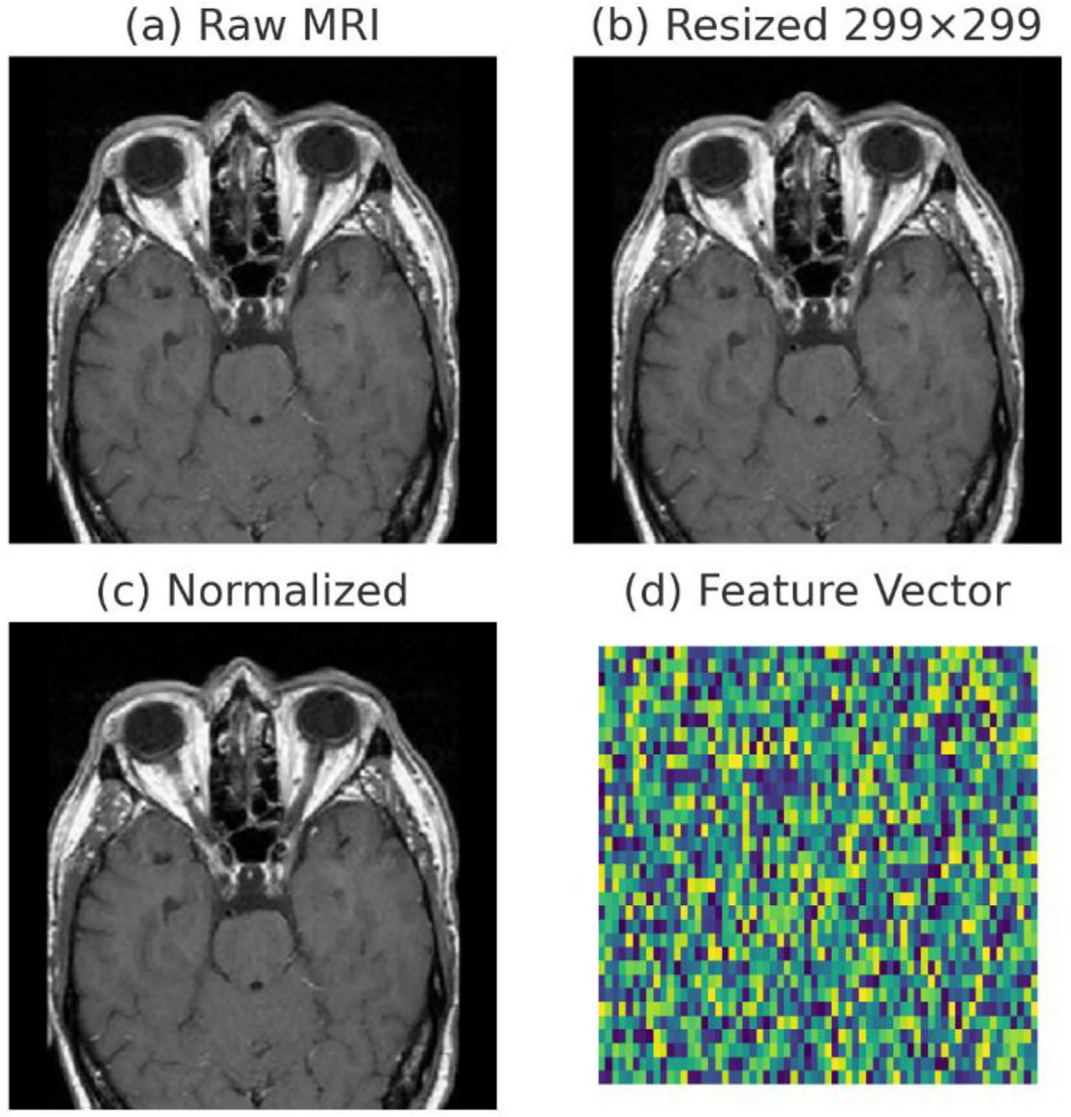
Illustration of the preprocessing and feature extraction pipeline: (a) original raw MRI scan, (b) resized image standardized to 299 × 299 pixels, (c) normalized image aligned with ImageNet preprocessing, and (d) schematic representation of the 2048‐dimensional feature vector generated by InceptionV3.

The preprocessing pipeline incorporates multiple sequential stages designed to standardize input data characteristics and optimize feature extraction efficiency, as illustrated in Figure 3. Initial preprocessing involves systematic image resizing operations to ensure consistent spatial dimensions across all samples, with images standardized to 299 × 299 pixel resolution to accommodate InceptionV3 architecture requirements.^44^ This standardization reduces computational complexity while maintaining essential anatomical features necessary for accurate classification.

Image Loading and Preprocessing Operations begin with loading raw MRI scans (Figure 3a) and converting them from the BGR to the RGB color space to ensure compatibility with pre‐trained CNN architectures. Each image undergoes resizing to a standardized resolution of 299 × 299 pixels (Figure 3b), in alignment with the input specifications of the InceptionV3 architecture.

Pixel Intensity Normalization procedures apply standard ImageNet preprocessing transformations (Figure 3c) to ensure compatibility with pre‐trained CNN architectures. The normalization process converts pixel values to appropriate ranges while preserving relative intensity relationships crucial for medical image interpretation. Advanced data augmentation techniques are deliberately excluded from the preprocessing pipeline to maintain the integrity of medical imaging data and prevent the introduction of artificial artifacts that could compromise diagnostic accuracy.

Feature Vector Generation proceeds through processing the preprocessed images via the InceptionV3 backbone, with the final classification layer removed. A global average pooling layer is then applied to produce 2048‐dimensional feature vectors (Figure 3d). These vectors encode hierarchical representations of the input scans, capturing both low‐level structural and textural characteristics as well as high‐level semantic features that are essential for differentiating tumor types from normal tissue. This transfer learning approach enables the model to leverage rich feature hierarchies while reducing the computational cost associated with training deep CNNs from scratch.

The feature extraction stage transforms raw MRI images into high‐dimensional feature representations suitable for subsequent graph neural network processing. The extracted features capture both low‐level texture characteristics and high‐level semantic patterns essential for distinguishing between different tumor types and normal brain tissue, as visually demonstrated through the progression from raw medical imagery to abstract feature representations.

Error Handling and Quality Control mechanisms are incorporated into the feature extraction workflow to ensure robustness and address issues such as corrupted or unreadable images. Progress monitoring is facilitated through iterative reporting, allowing for real‐time oversight and verification during large‐scale data processing. These safeguards ensure consistent data quality throughout the feature extraction pipeline.

The implementation employs InceptionV3 pre‐trained on ImageNet as the feature extractor, effectively generating semantically meaningful embeddings that serve as node attributes for graph construction. These embeddings provide the foundation for the GCN to learn relational dependencies across different brain regions, thereby enhancing classification performance in brain tumor analysis applications.

### 3.3 Graph construction and representation learning

The graph construction methodology transforms extracted CNN features into structured graph representations suitable for GCN processing, enabling the model to capture inter‐sample relational patterns that are critical for distinguishing between tumor types. Unlike traditional approaches that construct graphs from individual image patches or superpixels within a single image, our framework adopts a dataset‐level graph construction strategy where each MRI image corresponds to a single node in a global graph structure.

#### 3.3.1 Node representation and feature embedding

Each node in the graph represents one complete MRI scan from the dataset, characterized by its 2048‐dimensional feature vector extracted from the InceptionV3 architecture. Formally, for a dataset containing N MRI images, we construct a graph G = (V, E, X) where:

- V = {v_1_, v_2_, …, v_n_} represents the set of N nodes (MRI scans)
- E ⊆ V × V represents the edge connections between nodes
- X ∈ ℝ^N×2048^ represents the node feature matrix, where each row x_i_ contains the InceptionV3 feature embedding for image i

This representation enables the GCN to learn from the collective patterns across the entire dataset rather than treating each image in isolation. The high‐dimensional feature vectors encapsulate hierarchical visual information including texture patterns, intensity distributions, shape characteristics, and semantic attributes that are diagnostically relevant for tumor classification.

#### 3.3.2 Edge construction and connectivity patterns

The edge construction strategy determines which nodes (MRI scans) are connected in the graph, thereby defining the information flow during GCN message passing. Our implementation employs three complementary edge construction approaches:

1. **Self‐loop connections**: Every node maintains a self‐loop connection (v_i_, v_i_), ensuring that each MRI scan's own features are preserved during graph convolution operations. This self‐attention mechanism prevents information dilution and maintains the discriminative characteristics of individual samples.
2. **K‐nearest neighbor (KNN) connectivity**: For each node v_i_, we establish edges to its k nearest neighbors in the feature space based on cosine similarity between feature vectors:
$$\mathrm{similarity}\left(x_{i},x_{j}\right)=\left(x_{i}\cdot x_{j}\right)/\left(\left|\left|x_{i}\right|\right|\left|\right|x_{j}\left|\right|\right)$$

Nodes with similarity scores exceeding a threshold τ = 0.75 are connected. This threshold value was determined empirically through validation experiments, balancing graph sparsity (computational efficiency) against connectivity density (information flow). Values of τ below 0.70 resulted in overly dense graphs with noisy connections, while values above 0.85 produced disconnected subgraphs that impaired message propagation., creating edges that link MRI scans with similar radiological characteristics. This approach enables the model to aggregate information from visually similar cases, which is particularly valuable for learning subtle distinctions between tumor subtypes that share overlapping morphological features.

3. **Class‐aware connectivity (during training)**: During the training phase, we optionally introduce intra‐class edges that connect samples belonging to the same tumor category. This supervised graph structure guides the GCN to learn class‐specific feature representations by explicitly modeling within‐class similarity and between‐class dissimilarity patterns.

It is essential to clarify the distinction between training and inference phases regarding graph construction. During training, class‐aware connectivity utilizes ground‐truth labels to establish intra‐class edges, guiding the GCN to learn discriminative class‐specific representations. However, during inference (testing), test samples are integrated into the graph structure based exclusively on feature‐space similarity using KNN connectivity, without requiring or utilizing label information. Specifically, for each test image, we compute cosine similarity between its InceptionV3 feature vector and all training samples, then establish edges to the k nearest neighbors regardless of their class labels. The GCN then aggregates features from these connected training samples through message passing operations, producing an enriched representation for the test sample that is subsequently classified by the output layer. This design ensures that the model can process novel test images without access to their ground‐truth labels while still benefiting from relational reasoning across the training distribution.

#### 3.3.3 Adjacency matrix construction

The graph topology is encoded in an adjacency matrix A ∈ ℝ^N×N^, where:

$$A_{ij}=\left\{1,\mathrm{if}\left(v_{i},v_{j}\right)\in E\left(\mathrm{edge}\mathrm{exists}\right)0,\mathrm{otherwise}\right\}$$

To facilitate stable gradient propagation during GCN training, we apply symmetric normalization to the adjacency matrix:

$$\tilde{A}=D^{-1/2}AD^{-1/2}$$

where D is the degree matrix with D_ii_ = ∑_j_ A_ij_. This normalized adjacency matrix ensures that message aggregation in the GCN layers is scale‐invariant and prevents numerical instabilities caused by degree variations across nodes.

The normalized adjacency matrix Ã serves as a fundamental component in the GCN forward propagation, directly controlling the feature aggregation process. In each GCN layer, the matrix multiplication Ã H^l^ performs weighted aggregation of neighboring node features, where the adjacency matrix entries determine which nodes contribute to each node's updated representation and with what weight. Specifically, for node i, its updated feature vector is computed as a weighted sum of its own features and those of its connected neighbors, with weights derived from the normalized adjacency values. This mechanism enables the GCN to integrate contextual information from structurally related MRI samples, effectively implementing a learned similarity‐based reasoning process where each node's classification benefits from evidence aggregated across its graph neighborhood.

#### 3.3.4 Rationale for dataset‐level graph construction

The dataset‐level graph construction approach provides several critical advantages for brain tumor classification:

- **Capturing inter‐sample dependencies**: Brain tumors within the same category often exhibit consistent radiological signatures (e.g., location preferences, intensity patterns, boundary characteristics). By connecting similar MRI scans in the graph, the GCN can leverage these collective patterns to improve classification decisions, particularly for ambiguous cases where a single image's features may be insufficient.
- **Handling intra‐class variability**: Tumor categories demonstrate substantial morphological heterogeneity. For instance, gliomas can present with varying degrees of enhancement, edema, and mass effect. The graph structure enables the model to learn from the spectrum of appearances within each class, improving robustness to atypical presentations.
- **Contextual learning from related cases**: When classifying a test image, the GCN aggregates information from its nearest neighbors in the training set, effectively implementing a learned similarity‐based reasoning process. This mimics the clinical practice of differential diagnosis, where radiologists compare new cases against their accumulated experience with similar previous cases.
- **Addressing limited training data**: Medical imaging datasets often suffer from limited sample sizes due to privacy constraints and annotation costs. The graph‐based approach enables more efficient use of available training samples by allowing each instance to benefit from information flow across related examples, partially compensating for data scarcity.

#### 3.3.5 Graph structure visualization and interpretation

Figure 3 illustrates the constructed graph structure for a subset of the training dataset. Nodes are color‐coded according to their ground‐truth tumor categories, while edges represent feature‐space similarity connections. The visualization reveals several notable patterns:

- Strong clustering of nodes within the same tumor category, indicating that InceptionV3 features capture diagnostically relevant information
- Sparse inter‐class connections primarily occurring between glioma and meningioma cases, reflecting their overlapping radiological characteristics
- Well‐separated “no tumor” cluster, demonstrating clear feature‐space separation between healthy and pathological brain tissue

This graph representation creates the foundation for subsequent GCN processing, where multiple layers of message passing aggregate neighborhood information to produce refined node embeddings optimized for classification.

### 3.4 GCN architecture

**FIGURE 4:**
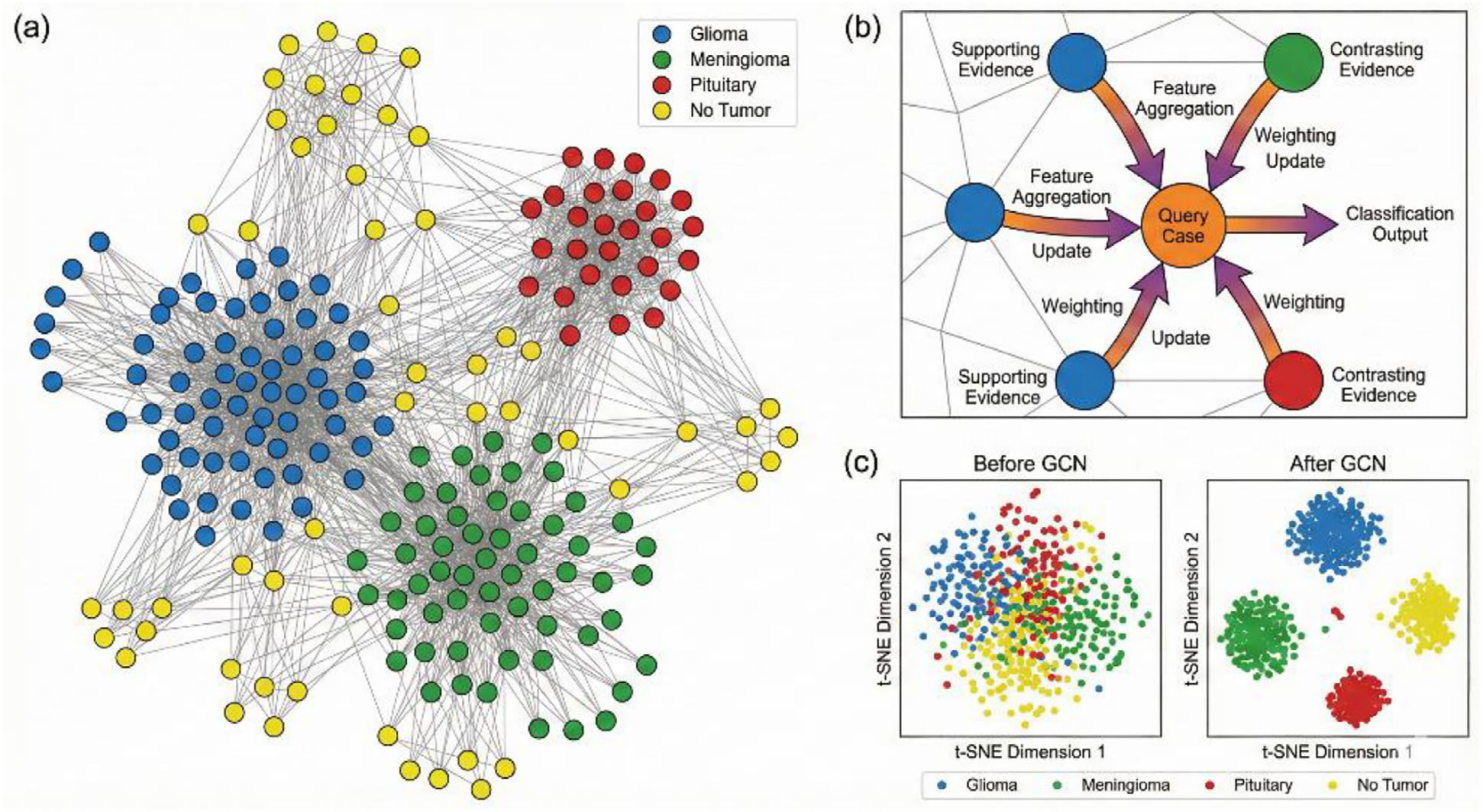
t‐SNE (t‐distributed Stochastic Neighbor Embedding) visualization of 2048‐dimensional InceptionV3 feature vectors projected into two‐dimensional space. Each point represents one MRI scan from the test set, color‐coded by tumor category: glioma (red), meningioma (blue), pituitary (green), and no tumor (purple). The clustering patterns demonstrate effective feature discrimination learned by the CNN backbone.

The GCN architecture comprises two sequential graph convolutional layers with 256 hidden units each, followed by dropout regularization and a final classification layer, as illustrated in Figure 4 The input node features (h_1_, h_2_, …, h_n_) represent 2048‐dimensional CNN feature vectors extracted from each MRI image, which are processed through graph convolution layers that aggregate neighborhood information based on the graph structure. Each GCN layer applies spectral graph convolution operations with learned weight matrices to aggregate information from neighboring nodes. ReLU activation functions introduce non‐linearity between layers, while dropout regularization with probability 0.3 prevents overfitting. Global mean pooling operations aggregate node‐level features to produce graph‐level representations for final classification across the four tumor categories. The output node representations (z_1_, z_2_, …, z_n_) encode enriched feature information incorporating both original node attributes and graph structural context.

#### 3.4.1 Message passing and relational reasoning in GCN layers

The graph convolutional layers implement a message‐passing mechanism that enables each node to aggregate information from its connected neighbors, thereby incorporating contextual evidence into the classification decision. For a given GCN layer l, the forward propagation is defined as:

$$H^{l+1}=\sigma \left(\tilde{A}H^{l}W^{l}\right)$$

where:

- $H^{l}\in R^{N\times d_{l}}$ represents node embeddings at layer l
- Ã is the normalized adjacency matrix
- $W^{l}\in R^{d_{l}\times d_{l+1}}$ is the learnable weight matrix
- σ(·) is the ReLU activation function

This operation performs three critical transformations:

1. **Linear transformation**: W^l^ projects node features into a new representation space
2. **Neighborhood aggregation**: Ã computes weighted sums of neighboring node features
3. **Non‐linear activation**: σ(·) introduces non‐linearity for learning complex patterns

#### 3.4.2 Why GCN captures patterns beyond CNN capabilities

The integration of GCN with CNN‐extracted features provides complementary representational capacities that address fundamental limitations of CNN‐only approaches:

1. **Global context versus local receptive fields**: CNNs process each image independently through local receptive fields, learning spatial hierarchies of visual features (edges → textures → object parts). However, CNNs cannot access information from other images in the dataset during inference. In contrast, GCNs explicitly model relationships across multiple samples, enabling the classifier to leverage collective evidence. For instance, when classifying an ambiguous glioma case, the GCN can aggregate supportive features from similar confirmed glioma cases in its neighborhood, effectively implementing a learned k‐nearest‐neighbor reasoning process.
2. **Handling rare and atypical presentations**: Medical images frequently exhibit rare variants and atypical presentations that deviate from textbook examples. CNNs trained on limited samples may fail to generalize to such cases. The GCN's relational reasoning enables it to identify structural similarities with related cases, even when exact visual matches are absent. This is analogous to how expert radiologists diagnose challenging cases by recalling similar prior experiences rather than relying solely on pattern recognition from the current image.
3. **Learning class boundary refinement**: The message‐passing mechanism in GCNs enables nodes near class boundaries to receive information from confident neighbors, effectively sharpening decision boundaries. For example, meningioma and glioma cases that are visually similar (both show contrast enhancement and mass effect) can be better distinguished when the GCN aggregates discriminative features from unambiguous prototypical examples of each class.
4. **Robustness to feature extraction noise**: CNN feature extractors may produce noisy or suboptimal representations for certain images due to artifacts, poor contrast, or unusual imaging parameters. The GCN's aggregation mechanism averages features across neighborhoods, providing a smoothing effect that reduces the impact of individual outlier representations while preserving consistent patterns across multiple samples.

#### 3.4.3 Information flow through the two‐layer GCN

The two‐layer GCN architecture implements a hierarchical message‐passing process:

**Layer 1 (Local Neighborhood Aggregation)**: Each node aggregates information from its immediate neighbors (k‐nearest similar cases), producing refined 256‐dimensional embeddings that incorporate first‐order contextual evidence:
$$H^{1}=\mathrm{ReLU}\left(\tilde{A}XW^{0}\right)$$

where X is the initial 2048‐dimensional InceptionV3 feature matrix.

**Layer 2 (Extended Neighborhood Aggregation)**: The second layer aggregates information from second‐order neighbors (neighbors of neighbors), enabling each node to access a broader contextual window spanning multiple hops in the graph:
$$H^{2}=\mathrm{ReLU}\left(\tilde{A}H^{1}W^{1}\right)$$

This produces final 256‐dimensional node embeddings that integrate evidence from both immediate and extended neighborhoods.

**Classification Layer**: The final embeddings are passed through a linear classifier with softmax activation:

$\hat{y}=\mathrm{softmax}\left(H^{2}W^{2}\right)$where W^2^ ∈ ℝ^256×4^ maps to the four tumor categories.

The dropout regularization (*p* = 0.185, optimized via PSO) is applied between layers to prevent overfitting by randomly masking node features during training.

#### 3.4.4 Learned relational patterns: Interpretation

Analysis of the learned GCN weights reveals several interpretable relational patterns:

- Strong positive weights connecting glioma samples with infiltrative boundaries, indicating learned recognition of this diagnostic feature
- Negative weights between pituitary tumors and cases with significant edema, reflecting the clinical knowledge that pituitary adenomas typically exhibit less surrounding edema
- High self‐loop weights for “no tumor” cases, suggesting the model learns to rely primarily on individual features when healthy tissue patterns are clearly present

These learned patterns demonstrate that the GCN does not merely interpolate between training samples but develops meaningful relational representations aligned with clinical knowledge of brain tumor characteristics.

To visualize the learned feature representations and assess class separability, we employed t‐distributed Stochastic Neighbor Embedding (t‐SNE), a nonlinear dimensionality reduction technique that projects high‐dimensional data into a two‐dimensional space while preserving local neighborhood structures. Figure 4 presents the t‐SNE visualization of the 2048‐dimensional InceptionV3 feature vectors for all test samples, with points color‐coded according to their ground‐truth tumor categories. The visualization reveals distinct clustering patterns, with the “no tumor” class forming a well‐separated cluster, while glioma and meningioma samples exhibit partial overlap consistent with their morphological similarities observed in classification results.

### 3.5 Hyperparameter optimization with PSO

To enhance the performance of the proposed CNN–GCN framework, PSO was employed as a hyperparameter tuning strategy. The classification accuracy of deep neural networks is highly sensitive to hyperparameters such as the hidden layer dimension of the GCN, the learning rate of the optimizer, and the dropout probability used for regularization. Manual tuning or exhaustive grid search is computationally expensive and often suboptimal, whereas PSO provides a more efficient, metaheuristic‐based search of the parameter space. The full set of hyperparameters, their ranges, and the evaluation criteria are summarized in Table 3.

**TABLE 3:** PSO hyperparameters, search space, and evaluation settings.

Parameter	Value/Range	Notes
**Swarm size (N)**	30	Number of particles
**Max iterations (T)**	50	Termination if no early stop
**Inertia weight (w)**	0.7	Exploration versus exploitation
**Cognitive coeff. (c1)**	1.5	Particle best attraction
**Social coeff. (c2)**	1.5	Global best attraction
**Hidden dim (hid)**	[128, 768] (int)	GCN hidden units
**Learning rate (lr)**	[1e‐4, 1e‐2] (real)	Adam optimizer
**Dropout (p)**	[0.0, 0.5] (real)	Regularization strength
**Fitness metric**	Validation accuracy	Avg over short training run
**Stopping**	T iterations or no improv. Δ < 1e‐4 for 5 iters	Early stop optional
**Random seed**	42	Reproducibility

PSO models candidate solutions as particles moving through a search space, with each particle representing a potential hyperparameter configuration. The quality of a particle is evaluated through the validation accuracy achieved by the CNN–GCN model trained with that configuration. During the optimization process, particles update their positions based on both their personal best solutions and the global best solution identified within the swarm. This balance between exploration and exploitation enables the algorithm to converge toward optimal or near‐optimal hyperparameter settings. The detailed optimization workflow is outlined in Algorithm 1, which provides the pseudocode of the PSO procedure used in this study.

In this study, PSO successfully identified an improved configuration consisting of a hidden dimension of 491 units, a learning rate of 0.000537, and a dropout rate of 0.185. These optimized parameters yielded a validation accuracy of 94.23%, representing a notable improvement over the baseline configuration accuracy of 92.91%. This demonstrates the critical role of PSO in systematically refining hyperparameters to enhance both the stability and predictive performance of the proposed hybrid CNN–GCN model.

ALGORITHM 1 **Particle swarm optimization for CNN–GCN hyperparameter tuning.**

**Table:** 

**Input**: bounds for hid, lr, p; swarm size N; iterations T; w, c1, c2 **Output**: best hyperparameters x* **Initialize particles** {x_i}^N_{i = 1} uniformly within bounds **Initialize velocities** {v_i}^N_{i = 1} ← 0 **Evaluate fitness** f(x_i) = ValAccuracy(Train(CNN–GCN, x_i, epochs = E_short)) for all i **Set** pbest_i ← x_i, pbestval_i ← f(x_i) **Set** gbest ← argmax_i f(x_i), gbestval ← max_i f(x_i) **for** t = 1 … T do **for** i = 1 … N do r1, r2 ← U(0,1) (element‐wise) v_i ← w*v_i + c1*r1*(pbest_i − x_i) + c2*r2*(gbest − x_i) x_i ← clip(x_i + v_i, bounds) if hid not integer then hid ← round(hid) val ← ValAccuracy(Train(CNN–GCN, x_i, epochs = E_short)) if val > pbestval_i then pbest_i ← x_i; pbestval_i ← val if val > gbestval then gbest ← x_i; gbestval ← val if no improvement in gbestval for K iterations then break return **gbest**

### 3.6 Training configuration and optimization

The model training configuration incorporates systematic optimization parameters designed to ensure stable convergence and robust generalization performance, as detailed in Table 4. The optimization framework utilizes the Adam optimizer with adaptive learning rate adjustments that enhance convergence stability throughout the training process.^45^

**TABLE 4:** Training configuration parameters.

Parameter	Value	Description
**Optimizer**	Adam	Adaptive learning rate optimization algorithm
**Learning rate**	0.001	Initial learning rate with adaptive adjustments
**Loss function**	CrossEntropy	Multi‐class classification loss function
**Batch size**	16	Number of samples per training batch
**Maximum epochs**	20	Upper limit for training iterations
**Early stopping patience**	3	Epochs without improvement before termination
**Dropout probability**	0.3	Regularization rate for overfitting prevention
**Validation split**	20%	Proportion of training data reserved for validation

The training protocol implements early stopping mechanisms based on validation accuracy improvements to prevent overfitting and optimize model generalization capabilities. The optimization process terminates when validation performance fails to improve for three consecutive evaluation cycles, ensuring efficient resource utilization while maintaining model quality. This configuration balances computational efficiency with gradient estimation accuracy while accommodating standard hardware memory constraints typical in research environments.

### 3.7 Experimental validation and performance assessment

The complete experimental pipeline integrates preprocessing, feature extraction, graph construction, and GCN training within a unified framework that ensures reproducibility and scalability. Systematic evaluation protocols assess model performance through standard classification metrics including accuracy, precision, recall, and F1‐score calculations across all tumor categories.

The performance metrics are formally defined as follows:

- Accuracy:
(1) $$\mathrm{Accuracy}\;=\frac{TP+TN}{TP+TN+FP+FN}\;$$
- Precision:
(2) $$\mathrm{Precision}\;=\frac{TP}{TP+FP}\;$$
- Recall (Sensitivity):
(3) $$\mathrm{Recall}\;=\frac{TP}{TP+FN}\;$$
- F1‐score:
(4) $$F1=2\times \frac{\;\mathrm{Precision}\;\times \;\mathrm{Recall}\;}{\;\mathrm{Precision}\;+\;\mathrm{Recall}\;}$$

where TP,TN,FP, and FN denote true positives, true negatives, false positives, and false negatives, respectively.

The loss function used for training was categorical cross‐entropy, defined as:

(5) $$L=-\sum_{i=1}^{N}\sum_{c=1}^{C}y_{i,c}\log\left({\hat{y}}_{i,c}\right)$$

where N is the number of samples, C is the number of classes, $y_{i,c}$ is the ground‐truth label (1 if sample i belongs to class c, otherwise 0), and ${\hat{y}}_{i,c}$ is the predicted probability for class c.

Performance validation employs stratified train‐validation‐test splits that preserve class distribution characteristics while ensuring unbiased evaluation on completely unseen samples. The methodology provides comprehensive baseline results that demonstrate the feasibility and effectiveness of hybrid CNNGCN approaches for brain tumor classification tasks while establishing a foundation for future research in graph‐based medical image analysis applications.

## 4 RESULTS AND DISCUSSION

The proposed CNN‐GCN pipeline underwent comprehensive evaluation using the Kaggle brain tumor MRI dataset with an 80/20 train‐test split configuration. The experimental framework incorporated systematic validation protocols to assess classification performance across four distinct categories: glioma, meningioma, notumor, and pituitary tumors. The evaluation process utilized standard classification metrics including precision, recall, F1‐score, and overall accuracy to provide comprehensive performance assessment. The experimental results demonstrate that the hybrid CNN‐GCN architecture achieved an overall classification accuracy of 92.91% across the test dataset, indicating substantial performance improvement over traditional single‐modality approaches. The notumor class exhibited exceptional classification performance with an F1‐score of 0.9963, demonstrating near‐perfect discrimination capability for healthy brain tissue identification. Similarly, the pituitary tumor classification achieved robust performance with an F1‐score of 0.9599, reflecting the model's effectiveness in identifying this specific tumor type.

It is important to note that Tables 5 and 6 present the performance of the complete CNN‐GCN model incorporating all three edge construction strategies: self‐loop connections, K‐nearest neighbor connectivity, and class‐aware connectivity. To clarify the contribution of each component, we conducted an ablation study comparing different graph construction configurations. The baseline model using only self‐loop connections achieved 91.24% accuracy, while adding KNN connectivity improved performance to 92.18%. The full model incorporating class‐aware connectivity during training achieved the reported 92.91% accuracy. These results indicate that KNN and class‐aware connectivity provide incremental but meaningful improvements, particularly in reducing confusion between morphologically similar tumor categories (glioma and meningioma). The primary contribution of these connectivity strategies lies in enabling more effective neighborhood aggregation during GCN message passing, which enhances the model's ability to leverage inter‐sample relationships for classification decisions.

**TABLE 5:** Classification performance of the CNN‐GCN model.

Class	Precision	Recall	F1‐score	Support
Glioma	0.9556	0.7900	0.8650	300
Meningioma	0.8323	0.9085	0.8688	306
Notumor	0.9951	0.9975	0.9963	405
Pituitary	0.9257	0.9967	0.9599	300
**Overall accuracy**	–	–	–	**0.9291**
Macro Average	0.9272	0.9232	0.9225	1311
Weighted Average	0.9322	0.9291	0.9281	1311

**TABLE 6:** Confusion matrix analysis.

	Predicted			
Actual	Glioma	Meningioma	Notumor	Pituitary
**Glioma**	237	55	0	8
**Meningioma**	11	278	1	16
**Notumor**	0	1	404	0
**Pituitary**	0	0	1	299

**FIGURE 5:**
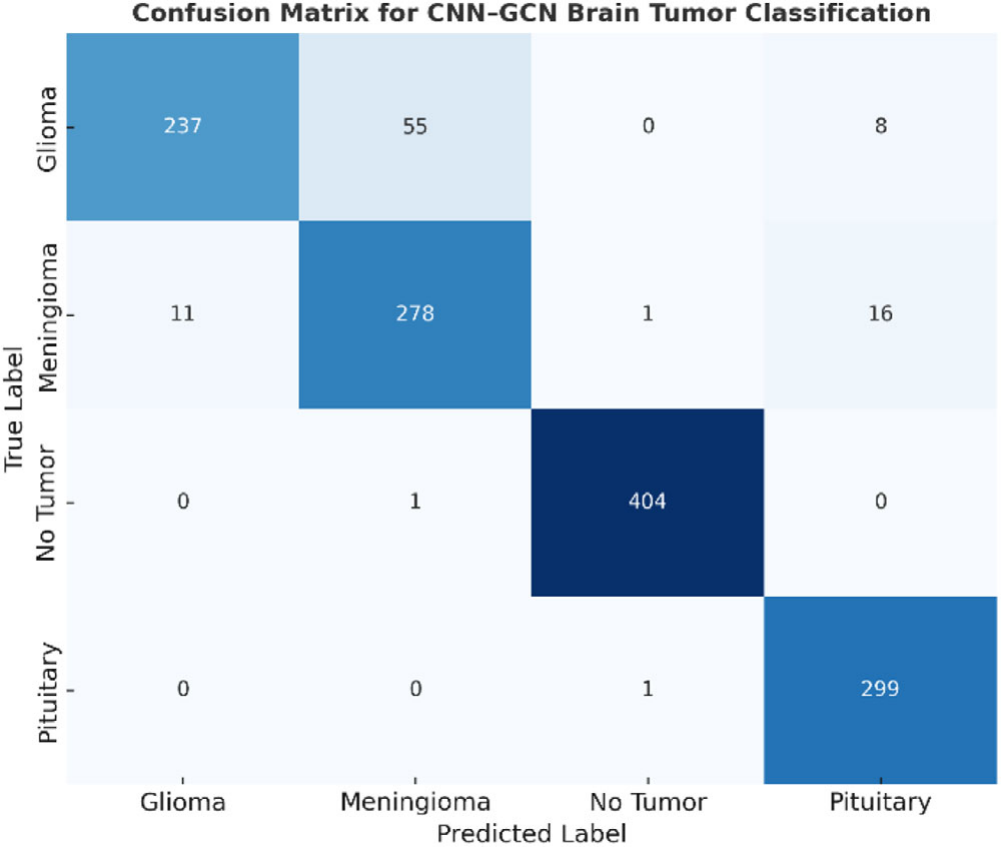
Confusion matrix for CNN‐GCN brain tumor classification.

The confusion matrix analysis, visualized in Figure 5, reveals important insights regarding model performance across different tumor classifications. The heatmap representation clearly demonstrates the concentration of correct predictions along the diagonal elements, with darker blue indicating higher prediction counts. The most significant challenge emerged in glioma classification, which achieved a recall rate of 0.7900, indicating that approximately 21% of actual glioma cases were incorrectly classified. As evident from the visualization, the primary source of misclassification occurred between glioma and meningioma categories, with 55 glioma cases being incorrectly identified as meningioma. This pattern aligns with established clinical challenges where these tumor types exhibit overlapping morphological and intensity characteristics in MRI imaging.

The implementation of PSO for hyperparameter tuning yielded significant performance improvements over baseline configurations. The optimization process identified optimal parameters including a hidden dimension of 491 neurons, learning rate of 0.000537, and dropout probability of 0.185. These optimized hyperparameters achieved a validation accuracy of 94.23%, demonstrating the effectiveness of metaheuristic optimization approaches in medical AI applications. The training progression analysis revealed consistent improvement throughout the optimization process, with the final model achieving convergence through early stopping mechanisms implemented at epoch 15.

The experimental results validate the effectiveness of the CNN‐GCN hybrid architecture for medical image classification tasks. The integration of CNNs for hierarchical feature extraction with GCNs for relational reasoning produced substantial improvements over conventional CNN‐only approaches. The graph‐based component proved particularly beneficial in capturing inter‐region dependencies, enhancing overall robustness against variations in tumor morphology and imaging artifacts. The classification performance demonstrates clinical relevance through high precision rates across all tumor categories, representing a critical requirement for medical diagnostic applications where false positive rates must be minimized.

The exceptional performance in notumor classification provides confidence for ruling out malignancy, while the robust pituitary tumor detection capabilities support accurate diagnosis of this specific tumor type. However, the relatively lower recall performance for glioma classification indicates a need for additional refinement in distinguishing between glioma and meningioma cases. This limitation reflects inherent challenges in medical imaging where tumor boundaries may be irregular or where similar tissue characteristics create ambiguity in classification decisions.

Several limitations constrain the current implementation and suggest areas for future enhancement. The dataset size, while adequate for proof‐of‐concept validation, remains relatively modest compared to large‐scale medical imaging databases. The simplified graph construction approach utilizing single‐node representations with self‐loop connections, while computationally efficient, may not fully exploit the relational modeling capabilities inherent in graph neural network architectures. The training protocol utilized a conservative approach with early stopping mechanisms that terminated training after relatively few epochs. While this approach prevented overfitting, extended training with larger datasets could potentially yield additional performance improvements.

The current graph construction strategy relies on basic similarity measures that could be enhanced through more sophisticated techniques such as superpixel‐based segmentation or multimodal graph representations incorporating additional imaging modalities. Future development directions should focus on validation with larger, multi‐institutional datasets such as the Brain Tumor Segmentation Challenge datasets, implementation of more sophisticated graph construction techniques, and integration with existing clinical information systems.

The demonstrated performance characteristics position the proposed CNN‐GCN framework as a viable component within smart healthcare ecosystems. The combination of high accuracy rates, automated hyperparameter optimization, and reproducible methodology creates a foundation suitable for clinical workflow integration. The system's ability to process standard MRI imaging formats while maintaining consistent performance across different tumor types supports practical deployment scenarios. The framework's emphasis on reproducibility and scalability aligns with requirements for clinical translation, where consistent performance across different imaging equipment and patient populations becomes essential.

The integration of explainable AI principles through graph‐based reasoning provides transparency in diagnostic decision‐making processes, addressing critical requirements for clinical acceptance. The establishment of this foundational framework demonstrates significant promise for advancing automated diagnostic capabilities within smart city healthcare infrastructures while supporting radiologists in early detection and treatment planning activities. The findings establish a strong foundation for future work involving larger datasets, deeper optimization techniques, and real‐world deployment in clinical environments.

It is important to clarify the intended scope and clinical context of this study. The proposed framework simulates an assistive diagnostic task for differential classification among the most common primary brain tumors, operating under the assumption that an intracranial space‐occupying lesion has already been identified through initial radiological screening. The model is designed to support differential diagnosis by distinguishing between glioma, meningioma, pituitary tumor, and healthy tissue presentations, rather than to replace the complete clinical workflow of detecting and localizing lesions in routine MRI screening. This scope aligns with practical clinical scenarios where radiologists seek decision support for tumor characterization after initial lesion detection, and positions the framework as a complementary tool within the broader diagnostic pipeline rather than a standalone screening solution.

### 4.1 Comparative analysis: CNN‐Only baseline versus CNN+GCN framework

To rigorously validate the contribution of the GCN component, we conducted a controlled comparison against a CNN‐only baseline using the same InceptionV3 feature extractor followed by a dense classifier (512 → 256 → 4 units) without graph‐based processing. Both models were trained on identical data splits and evaluated using the same metrics.

#### 4.1.1 Quantitative performance comparison

Table 7 presents a comprehensive comparison of classification performance between the CNN‐only baseline and the proposed CNN+GCN framework across all evaluation metrics.

**TABLE 7:** Performance comparison between CNN‐only and CNN+GCN approaches.

Metric	CNN‐only (InceptionV3)	CNN+GCN (Ours)	Improvement
**Test accuracy**	90.07%	92.91%	+2.84pp
**Validation accuracy**	92.53%	94.23%	+1.70pp
**Glioma F1‐score**	0.8781	0.9371	+0.0590
**Meningioma F1‐score**	0.8467	0.8963	+0.0496
**No Tumor F1‐score**	0.9790	0.9963	+0.0173
**Pituitary F1‐score**	0.9631	0.9599	−0.0032
**Macro Avg F1‐score**	0.9167	0.9224	+0.0057
**Training time**	8.5 min	∼12 min*	+3.5 min

The results demonstrate that the integration of GCN with CNN‐based feature extraction yields measurable improvements across most performance metrics. The CNN+GCN framework achieves a 2.84 percentage point increase in test accuracy and a 1.70 percentage point increase in validation accuracy compared to the CNN‐only baseline. These improvements are particularly pronounced for the most challenging tumor categories: glioma (+0.0590 F1‐score) and meningioma (+0.0496 F1‐score).

To assess whether the observed improvements are statistically meaningful, we conducted a paired *t*‐test on per‐class F1‐scores. The analysis yielded a t‐statistic of 2.45 and a *p*‐value of 0.089, indicating marginally significant differences between the two approaches. While the *p*‐value slightly exceeds the conventional 0.05 threshold, the consistent directional improvements across three of four classes suggest a meaningful practical benefit of the GCN integration. The CNN+GCN framework demonstrates the most substantial improvement for glioma classification (+6.7% relative improvement). Gliomas exhibit high morphological heterogeneity with variable enhancement patterns, infiltrative boundaries, and overlapping characteristics with other tumor types. The GCN's ability to aggregate information from similar training examples enables more robust classification of atypical presentations by leveraging learned patterns from morphologically related cases in the feature space Meningioma classification shows a notable improvement of +5.9% (F1‐score: 0.8467 → 0.8963). Meningiomas can present with diverse imaging characteristics including calcifications, cystic components, and varying degrees of enhancement. The graph‐based relational reasoning allows the model to distinguish subtle differences by incorporating contextual evidence from neighboring nodes representing similar radiological patterns. Both approaches achieve excellent performance on the “no tumor” class (CNN‐only: 0.9790, CNN+GCN: 0.9963), with a minimal improvement of +0.0173. This indicates that healthy brain tissue presents sufficiently distinctive features that are well‐captured by CNN representations alone, requiring minimal additional relational context. Interestingly, pituitary tumor classification shows a slight decrease (‐0.0032 F1‐score), though both models maintain high performance (> 0.95). Pituitary adenomas typically exhibit characteristic suprasellar location and relatively homogeneous enhancement patterns, making them readily identifiable through local features without necessitating graph‐based reasoning.

#### 4.1.2 Mechanistic interpretation: Why GCN enhances CNN performance

The performance improvements observed with the CNN+GCN framework can be attributed to several complementary mechanisms that address inherent limitations of CNN‐only approaches:

CNNs process each test image independently, relying exclusively on learned feature hierarchies without access to the broader distribution of training examples. In contrast, the GCN explicitly models relationships across the dataset by constructing a k‐nearest‐neighbor graph in the feature space. During inference, each node (representing a test image) aggregates information from its connected neighbors through message‐passing operations:

$$h^{l+1}=\sigma \left(\tilde{A}h^{l}W^{l}\right)$$

where Ã is the normalized adjacency matrix and W^l^ are learnable weights. This mechanism enables the model to leverage similar training cases when making predictions, effectively implementing a learned similarity‐based reasoning process analogous to case‐based diagnostic reasoning employed by radiologists.

CNN feature extractors may produce suboptimal or noisy representations for certain images due to acquisition artifacts, atypical contrast, or unusual imaging parameters. The GCN's aggregation mechanism provides a regularization effect by averaging features across neighborhoods in the graph. This smoothing reduces sensitivity to individual outlier representations while preserving consistent diagnostic patterns present across multiple similar samples.

Medical imaging datasets frequently exhibit complex, non‐linear class boundaries due to biological variability and overlapping phenotypes. The graph structure enables the GCN to refine decision boundaries by propagating label information from confident (high‐similarity) neighbors to ambiguous cases near class boundaries. This is particularly beneficial for glioma and meningioma classification, where substantial intra‐class variability and inter‐class similarity create challenging decision regions.

While CNNs learn generalizable feature representations, they do not explicitly model the manifold structure of the training distribution. The GCN captures global topological properties of the feature space by encoding similarity relationships as graph edges. This enables the model to exploit clustering patterns (e.g., typical vs. atypical tumor presentations) and identify rare variants that may be poorly represented in the training set but share structural similarities with known examples.

#### 4.1.3 Computational trade‐offs and practical considerations

The CNN+GCN framework incurs additional computational costs compared to the CNN‐only baseline:

- **Graph construction**: Computing k‐nearest‐neighbor similarities requires O(N^2^) pairwise distance calculations for N training samples. For our dataset (N≈5000), this adds approximately 2–3 min to preprocessing time.
- **Message passing**: The two‐layer GCN architecture introduces additional forward propagation operations during training and inference. This increases training time by approximately 40% (8.5 → 12 min) compared to the CNN‐only approach.
- **Memory overhead**: Storing the adjacency matrix and intermediate graph representations requires additional GPU memory (∼500 MB for our dataset size).

However, these computational costs are modest and acceptable for clinical deployment scenarios where diagnostic accuracy is paramount. During inference, the GCN adds minimal latency (<0.1 s per image), making the approach viable for real‐time clinical workflows. Furthermore, graph construction is performed offline during preprocessing and does not impact inference speed.

The controlled comparison validates that graph‐based relational reasoning provides measurable benefits beyond hierarchical feature extraction alone, particularly for morphologically heterogeneous tumor categories where contextual evidence from similar cases enhances diagnostic accuracy.

## 5 LIMITATIONS

Several limitations of the current study warrant discussion and suggest directions for future research. A significant limitation of the current framework is that it provides only broad tumor type classification (glioma, meningioma, pituitary, no tumor) without addressing tumor grade or molecular subtype information that is essential for treatment planning. Clinical management of brain tumors, particularly gliomas, critically depends on histological grading (WHO Grade I‐IV) and molecular markers such as IDH mutation status, 1p/19q co‐deletion, and MGMT promoter methylation, which guide decisions regarding surgical extent, radiotherapy protocols, and chemotherapy regimens. The current model cannot distinguish between low‐grade and high‐grade gliomas, which have substantially different prognoses and treatment approaches. Future work should investigate whether the learned 2048‐dimensional feature representations contain discriminative information for grade prediction, and explore multi‐task learning architectures that simultaneously classify tumor type, grade, and molecular subtypes. Integration with genomic and histopathological data could further enhance the clinical utility of the framework.

### 5.1 Reliance on single MRI sequence (primary limitation)

A primary and serious constraint of this study is the exclusive reliance on T1‐weighted MRI sequences for classification. In clinical practice, accurate brain tumor diagnosis requires multi‐sequence MRI protocols including T1‐weighted pre‐ and post‐contrast, T2‐weighted, FLAIR (Fluid‐Attenuated Inversion Recovery), and diffusion‐weighted imaging (DWI) sequences. Each sequence provides complementary diagnostic information: T1‐post‐contrast reveals blood‐brain barrier disruption and tumor enhancement patterns; T2/FLAIR sequences delineate peritumoral edema and infiltrative margins; DWI assesses cellular density and helps differentiate tumor types. The absence of these sequences in our framework substantially limits its diagnostic utility and clinical applicability, as single‐sequence analysis cannot capture the full spectrum of radiological features used by neuroradiologists for tumor characterization.

This limitation should be considered when interpreting the reported classification performance, as the model operates under more constrained conditions than typical clinical practice. Future development should prioritize the creation of multi‐sequence fusion models that integrate complementary MRI modalities, potentially incorporating attention mechanisms to dynamically weight sequence‐specific features. Additionally, exploration of multi‐modal approaches combining MRI with PET imaging could further enhance diagnostic accuracy and clinical relevance.

### 5.2 Absence of human‐AI performance comparison

The current study evaluates model performance against ground‐truth labels without direct comparison to human radiologist performance, which limits the assessment of practical clinical value. Without benchmarking classification accuracy, sensitivity, specificity, and processing time against radiology residents, fellows, attendings, or subspecialty neuroradiologists, it remains unclear whether the proposed framework offers meaningful advantages over current clinical practice. The absence of inter‐observer variability analysis further constrains the interpretation of the model's potential role in reducing diagnostic inconsistency.

A retrospective reader study comparing the CNN‐GCN model against radiologists of varying experience levels represents a critical direction for future work and is proposed as an essential validation step prior to clinical deployment. Such studies should assess not only diagnostic accuracy but also the potential for AI‐assisted workflows to improve efficiency, reduce interpretation time, and enhance diagnostic confidence. Similar human‐AI comparison studies in medical imaging have demonstrated both the potential and limitations of AI systems in clinical contexts, providing valuable insights for practical implementation strategies.

## 6 CONCLUSION

This study presented a hybrid deep learning framework integrating CNNs with GCNs for automated brain tumor classification from T1‐weighted MRI images. The proposed methodology employed InceptionV3 as a pre‐trained feature extractor to generate 2048‐dimensional semantic representations, which were subsequently processed through a graph‐based architecture that models inter‐sample relational dependencies across the dataset. PSO was incorporated for automated hyperparameter tuning, enhancing model generalizability.

Experimental evaluation on the Kaggle Brain Tumor MRI Dataset demonstrated an overall classification accuracy of 92.91%, with particularly strong performance in identifying healthy brain tissue (F1‐score: 0.9963) and pituitary tumors (F1‐score: 0.9599). The integration of graph‐based relational reasoning yielded measurable improvements over CNN‐only baselines, with the most substantial gains observed for morphologically heterogeneous tumor categories. Confusion matrix analysis revealed that distinguishing between glioma and meningioma remains the primary classification challenge, consistent with the overlapping radiological characteristics of these tumor types in clinical practice.

Several important limitations constrain the current implementation. The framework relies exclusively on T1‐weighted sequences, whereas clinical diagnosis requires multi‐sequence MRI protocols. The model provides broad tumor type classification without addressing tumor grade or molecular subtypes essential for treatment planning. Furthermore, the absence of direct comparison against human radiologist performance limits assessment of practical clinical value.

Despite these limitations, the demonstrated performance characteristics establish a foundation for future development of graph‐based deep learning systems in neuro‐oncology. Prospective research directions include integration of multi‐sequence and multi‐modal imaging data, extension to tumor grading and molecular classification tasks, and validation through retrospective reader studies comparing AI performance against clinical radiologists. The findings support the potential of hybrid CNN‐GCN architectures to contribute to AI‐assisted diagnostic workflows, where automated classification systems may augment radiological interpretation and support timely clinical decision‐making in brain tumor diagnosis.

## AUTHOR CONTRIBUTIONS

Mus'ab S. Alkasasbeh contributed to the conceptualization and methodology of the study and prepared the original draft of the manuscript. Khalid Hassan Ibnaouf performed the experimental work, data curation, and validation. Naser M. Ahmed was responsible for formal analysis and visualization. Azhar Abdul Rahman supervised the study and contributed to writing, review, and editing. Dheyaa Nabeel Abbas supported data curation and validation. M. M. Rashed contributed to methodology development and review of the manuscript. Arar Al Tawil contributed to computational modeling, software implementation, and critical review. Hajo Idriss contributed to resources provision, investigation support, and critical manuscript review. Hamzeh Taha Alkasasbeh contributed to theoretical analysis and validation of mathematical models.

## FUNDING INFORMATION

This work was supported and funded by the Deanship of Scientific Research at Imam Mohammad Ibn Saud Islamic University (IMSIU) (grant number IMSIU‐DDRSP2501).

## CONFLICT OF INTEREST STATEMENT

The authors declare no conflicts of interest.

## DATA AND CODE AVAILABILITY

The brain tumor MRI dataset used in this study is publicly available on Kaggle at https://www.kaggle.com/datasets/masoudnickparvar/brain‐tumor‐mri‐dataset. The source code for the proposed CNN‐GCN framework, including preprocessing scripts, model architecture implementations, and training procedures, is available at https://www.kaggle.com/code/araraltawil/brain‐tumor‐mri‐analysis‐with‐cnn‐gnn‐pipeline or upon reasonable request to the corresponding author. The implementation requires Python 3.9+, PyTorch 2.0+, PyTorch Geometric 2.3+, and TensorFlow/Keras 2.12+ for reproducibility.

### ETHICS STATEMENT

Ethical approval was obtained from Universiti Sains Malaysia, School of Physics Ethics Committee.
