# Correction to “Hybrid CNN–GCN framework for brain tumor MRI classification: A graph‐based approach to smart healthcare diagnostics”

**DOI:** 10.1002/acm2.70596

**Published:** 2026-05-20

Alkasasbeh MS, Ibnaouf KH, Ahmed NM, et al. Hybrid CNN–GCN framework for brain tumor MRI classification: a graph‐based approach to smart healthcare diagnostics. *J Appl Clin Med Phys*. 2026;27:e70560. doi:10.1002/acm2.70560

The Funding Information section of the above article contained a typographical error in the grant number. The grant number was published as IMSIU‐DDRSP2501, whereas the correct grant number is IMSIU‐DDRSP2601. The corrected Funding Information statement should read as follows: “This work was supported and funded by the Deanship of Scientific Research at Imam Mohammad Ibn Saud Islamic University (IMSIU) (grant number IMSIU‐DDRSP2601).”

The authors apologize for this error and any inconvenience it may have caused.

